# Feasibility of Imaging EpCAM Expression in Ovarian Cancer Using Radiolabeled DARPin Ec1

**DOI:** 10.3390/ijms21093310

**Published:** 2020-05-07

**Authors:** Anzhelika Vorobyeva, Elena Konovalova, Tianqi Xu, Alexey Schulga, Mohamed Altai, Javad Garousi, Sara S. Rinne, Anna Orlova, Vladimir Tolmachev, Sergey Deyev

**Affiliations:** 1 Department of Immunology, Genetics and Pathology, Uppsala University, 751 85 Uppsala, Sweden; tianqi.xu@igp.uu.se (T.X.); mohamed.altai@igp.uu.se (M.A.); javad.garousi@igp.uu.se (J.G.); vladimir.tolmachev@igp.uu.se (V.T.); 2 Research Centrum for Oncotheranostics, Research School of Chemistry and Applied Biomedical Sciences, Tomsk Polytechnic University, 634050 Tomsk, Russia; schulga@gmail.com (A.S.); anna.orlova@ilk.uu.se (A.O.); 3 Molecular Immunology Laboratory, Shemyakin & Ovchinnikov Institute of Bioorganic Chemistry, Russian Academy of Sciences, 117997 Moscow, Russia; elena.ko.mail@gmail.com (E.K.); deyev@ibch.ru (S.D.); 4 Department of Medicinal Chemistry, Uppsala University, 751 23 Uppsala, Sweden; sara.rinne@ilk.uu.se; 5 Science for Life Laboratory, Uppsala University, 751 23 Uppsala, Sweden; 6 Bio-Nanophotonic Lab, Institute of Engineering Physics for Biomedicine (PhysBio), National Research Nuclear University ‘MEPhI’, 115409 Moscow, Russia; 7 Center of Biomedical Engineering, Sechenov University, 119991 Moscow, Russia

**Keywords:** EpCAM, radionuclide, molecular imaging, SPECT, iodine, PIB, ovarian, cancer

## Abstract

Epithelial cell adhesion molecule (EpCAM) is overexpressed in 55%–75% of ovarian carcinomas (OC). EpCAM might be used as a target for a treatment of disseminated OC. Designed ankyrin repeats protein (DARPin) Ec1 is a small (18 kDa) protein, which binds to EpCAM with subnanomolar affinity. We tested a hypothesis that Ec1 labeled with a non-residualizing label might serve as a companion imaging diagnostic for stratification of patients for EpCAM-targeting therapy. Ec1 was labeled with ^125^I using N-succinimidyl-*para*-iodobenzoate. Binding affinity, specificity, and cellular processing of [^125^I]I-PIB-Ec1 were evaluated using SKOV-3 and OVCAR-3 ovarian carcinoma cell lines. Biodistribution and tumor-targeting properties of [^125^I]I-PIB-Ec1 were studied in Balb/c nu/nu mice bearing SKOV-3 and OVCAR-3 xenografts. EpCAM-negative Ramos lymphoma xenografts served as specificity control. Binding of [^125^I]I-PIB-Ec1 to ovarian carcinoma cell lines was highly specific and had affinity in picomolar range. Slow internalization of [^125^I]I-PIB-Ec1 by OC cells confirmed utility of non-residualizing label for in vivo imaging. [^125^I]I-PIB-Ec1 provided 6 h after injection tumor-to-blood ratios of 30 ± 11 and 48 ± 12 for OVCAR-3 and SKOV-3 xenografts, respectively, and high contrast to other organs. Tumor targeting was highly specific. Saturation of tumor uptake at a high dose of Ec1 in SKOV-3 model provided a rationale for dose selection in further studies using therapeutic conjugates of Ec1 for targeted therapy. In conclusion, [^125^I]I-PIB-Ec1 is a promising agent for visualizing EpCAM expression in OC.

## 1. Introduction

Efficient diagnosis and treatment of disseminated ovarian cancer (OC) remains an unmet clinical need. Up to 85% of OC patients are diagnosed at advanced stages, when cancer has spread to the abdomen (stage III) or outside the abdomen and to liver (stage IV) [1]. The standard treatment, cytoreductive surgery in combination with chemotherapy, is inefficient at these stages. The lack of effective and safe systemic therapy options leads to fast progression of the cancer and only 10%–20% of these patients experience 10-year survival following standard treatment [1].

Targeting through molecular recognition of cancer-associated cell surface proteins is a way to increase treatment selectivity. A potential molecular target for OC is epithelial cell adhesion molecule (EpCAM). It is considered a pancarcinoma antigen due to its overexpression in many carcinomas [2,3]. It was found that in patients with OC overexpression of EpCAM was dependent on histology [3,4]. Mucinous OC had a 55% EpCAM overexpression rate, while serous, endometrioid, or other histologies had a rate of 76% [3]. EpCAM is an independent prognostic marker for reduced survival of patients with epithelial OC, particularly at stages III and IV [4]. Importantly, it was found that the EpCAM overexpression level is higher in metastatic and recurrent ovarian tumors compared to primary lesions [5]. These studies suggest that EpCAM might be a potential biomarker for evaluating the progression of OC and represent a promising molecular therapeutic target.

Several therapeutic approaches for targeting EpCAM in lung, gastric, colorectal, breast, and ovarian cancers have been evaluated in clinical trials [6,7,8]. Immunotherapy with the trifunctional anti-EpCAM x anti-CD3 antibody catumaxomab was approved in 2009 by the European Commission for the treatment of malignant ascites in cancer patients with EpCAM-expressing tumors. Intraperitoneal infusion of catumaxomab effectively reduced ascites and eliminated EpCAM-positive cancer cells in the ascites fluid of OC patients [9]. Adding an EpCAM-targeting immunotoxin MOC31PE to cytoreductive surgery in combination with hyperthermic intraperitoneal chemotherapy significantly increased the survival of patients with peritoneal metastases of colorectal cancer [10,11]. Moreover, preclinical studies demonstrated superior efficacy of MOC31PE in in vitro and in vivo models of ovarian cancer in comparison with cytotoxic drugs [12].

In order for a targeted therapy to be effective, the target expression level should be sufficient to enable delivery to and retention of a therapeutic agent at the tumor site. The issue of heterogeneity of EpCAM expression between patients and between tumors in the same patient [3,13] creates a need for selection of patients who would benefit from targeted therapy. A recent clinical trial evaluating EpCAM-targeted therapy used immunohistochemical staining of primary tumors to select patients [13]. However, the biopsy-based methods do not provide comprehensive information about target expression as they are limited in the number of samples that can be taken, locations and sizes of tumors. Radionuclide molecular imaging using positron emission tomography (PET) or single photon emission computed tomography (SPECT) is a non-invasive technique that provides whole-body information about target expression. In addition to enabling patient selection before therapy, it could be used for assessment of effectiveness of treatment, changes in target expression or receptor occupancy and could be performed repeatedly.

A common approach to diagnostic radionuclide imaging uses therapeutic monoclonal antibodies (mAbs) or their fragments. Several EpCAM-targeting imaging probes based on mAb scaffold (full-length IgG, F(ab’)2 and Fab fragments) were previously evaluated in clinics [14,15,16]. In 1998, the FDA approved a Fab fragment of the murine antibody NR-LU-10 labeled with technetium-99m, nofetumomab merpentan (Verluma), for staging of several carcinoma types, including ovarian [16].

Due to the large size, mAbs and their fragments have a long residence in blood, slow extravasation, and slow accumulation in tumors leading to limited sensitivity of imaging. The specificity of imaging could also be reduced due to enhanced permeability and retention (EPR) effect in tumors resulting in unspecific accumulation of bulky proteins. Decreasing the size of targeting proteins could overcome the limitations of mAbs and provide imaging at the day of injection [17]. Small engineered scaffold proteins (ESP), such as affibody molecules, ADAPTs, and designed ankyrin repeat proteins (DARPins), are attractive alternatives to antibodies for radionuclide-based imaging. Due to their rapid clearance from blood and normal tissues and high tumor accumulation, they provide high contrast imaging of tumors shortly after injection [17]. Clinical studies using affibody molecules have demonstrated high sensitivity of imaging of human epidermal growth factor receptor 2 (HER2)-expressing tumors and discrimination between high and low expression of HER2 [18,19].

DARPin scaffold is assembled from four to six helix-turn-helix repeats resulting in proteins with molecular weight from 14 to 18 kDa [20]. This scaffold is distinguished by a high thermal stability and resistance to proteases. DARPin combinatorial libraries were created by randomization of surface amino acids, which enabled selection of binders with subnanomolar affinity to several cancer-associated molecular targets [20,21]. DARPin-based imaging probes demonstrated excellent visualization of HER2 expression in human xenografts in mice within 4–6 h after injection [22,23,24,25].

Selection of several DARPins binding to EpCAM with high affinities were reported [26]. Preclinical studies demonstrated feasibility of the use of anti-EpCAM DARPin-toxin fusion protein for treatment of several types of cancer [27,28]. Recently, we have demonstrated that imaging of EpCAM expression in pancreatic cancer xenografts using radiolabeled DARPin Ec1 is feasible [29]. We have shown that internalization of Ec1 by pancreatic cancer cells is slow, while internalization by excretory organs was rapid. This permitted us to use a non-residualizing [^125^I]-*para*-iodobenzoate ([^125^I]I-PIB) label for Ec1. After internalization and lysosomal proteolysis of targeted proteins, lipophilic catabolites of non-residualizing label diffuse rapidly from cells [30]. This results in a rapid decrease of activity in liver and kidneys. In tumors, [^125^I]I-PIB-Ec1 remains predominantly bound to EpCAM on the surface of malignant cells for several hours, which results in better retention of activity in comparison with liver and kidney and better imaging contrast. Apparently, applicability of such approach is critically dependent on internalization rate of Ec1 after binding to EpCAM. DARPins are a new class of targeting probes, and it is not known if malignant cells of different origin have the same internalization pattern.

The primary goal of this study was to test a hypothesis that an internalization of Ec1/EpCAM complex by ovarian cancer cells is slow enough to permit imaging of tumors using [^125^I]I-PIB-Ec1 a few hours after injection. The secondary goal was to evaluate if EpCAM on ovarian cancer xenografts is sufficiently accessible to permit DARPin-mediated delivery of cytotoxic payload.

## 2. Results

### 2.1. Radiolabeling and Stability

**Figure 1:**
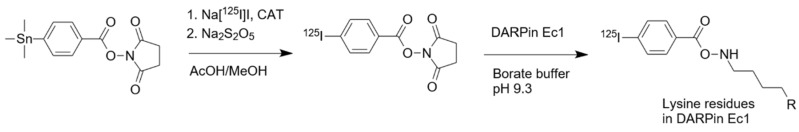
Radioiodination of designed ankyrin repeats protein (DARPin) Ec1 is a one-pot reaction achieved in two steps without intermediate purification. First, N-succinimidyl-*para*-(trimethylstannyl)benzoate is iodinated using Chloramine-T (CAT); in the second step, [^125^I]-*para*-iodobenzoate ([^125^I]I-PIB) is attached to lysine residues in DARPin Ec1.

Radioiodination of Ec1 using [^125^I]I-PIB (Figure 1) was performed as described previously [25,29] with 20% ± 5% (*n* = 3) radiochemical yield. Purification using size-exclusion NAP-5 column provided [^125^I]I-PIB-Ec1 with over 98% purity.

### 2.2. In Vitro Studies

**Figure 2:**
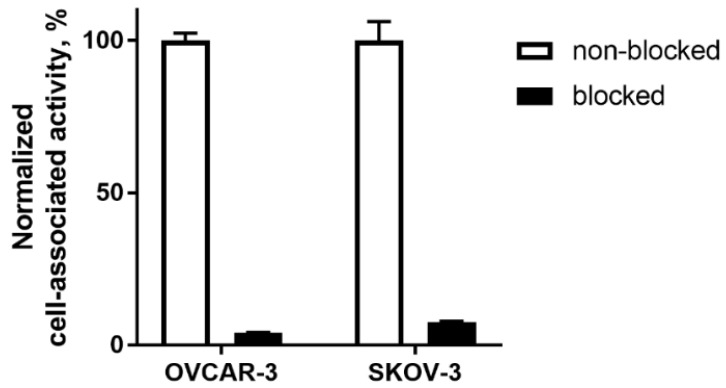
Binding specificity of [^125^I]I-PIB-Ec1 to epithelial cell adhesion molecule (EpCAM)-expressing OVCAR-3 and SKOV-3 ovarian cancer cells in vitro. For blocking, 100-fold molar excess of non-labeled Ec1 DARPin was added to blocked groups. Final concentration of radiolabeled compound was 2 nM. Data are presented as mean from three samples ± SD.

In vitro evaluation was performed using EpCAM-expressing OVCAR-3 and SKOV-3 ovarian cancer cells. To demonstrate binding specificity of [^125^I]I-PIB-Ec1 to EpCAM, the EpCAM receptors were saturated with 100-fold molar excess of non-labeled Ec1 before addition of the radiolabeled compound. Blocking the EpCAM receptors resulted in a significant (*p* < 0.001) decrease of [^125^I]I-PIB-Ec1 uptake (Figure 2).

**Figure 3:**
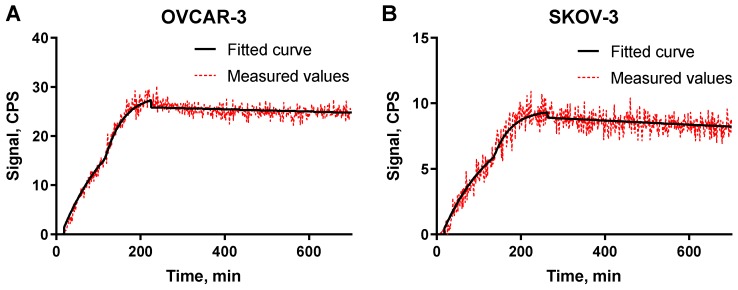
LigandTracer sensorgrams of [^125^I]I-PIB-Ec1 binding to (**A**) OVCAR-3 cells and to (**B**) SKOV-3 cells. The association was measured at 3 and 9 nM concentrations.

The binding kinetics of [^125^I]I-PIB-Ec1 to OVCAR-3 and SKOV-3 cells were measured using LigandTracer (Figure 3). Rapid binding and slow dissociation was observed for both cell lines. The K*_D_* values for both cell lines were in the picomolar range (Table 1).

**Figure 4:**
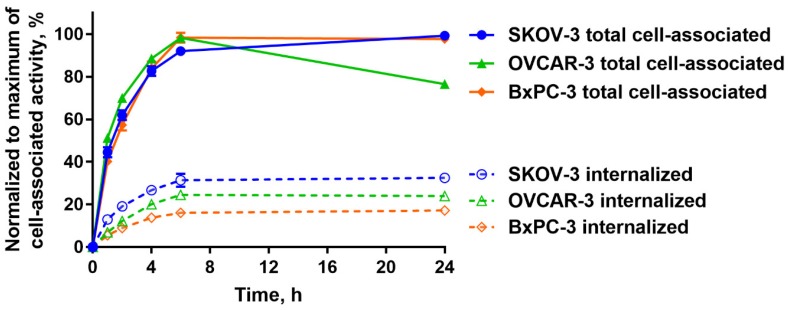
Cellular processing of [^125^I]I-PIB-Ec1 by OVCAR-3, SKOV-3 ovarian cancer cells, and BxPC-3 pancreatic cancer cells over 24 h continuous incubation. Data are shown as mean ± SD (*n* = 3); when error bars are smaller than symbols, they might not be visible.

A comparison of the processing of [^125^I]I-PIB-Ec1 by OVCAR-3 and SKOV-3 ovarian cancer cells and BxPC3 pancreatic cancer cells during continuous incubation is shown in Figure 4. A general pattern of [^125^I]I-PIB-Ec1 processing by a different cell line had a common feature, an increase of cell-associated activity was observed up to 6 h of incubation. Further fate of activity was somewhat different: the cell-associated activity was marginally increased for SKOV-3 cells, decreased by 25% for OVCAR-3, and remained approximately constant for BxPC-3. The internalized activity was the highest for SKOV-3 cells. Internalized fraction was higher for both ovarian cancer cell lines compared to BxPC-3 cells. Still, it was around 30% of cell-associated activity at 6 h.

### 2.3. In Vivo Studies

**Figure 5:**
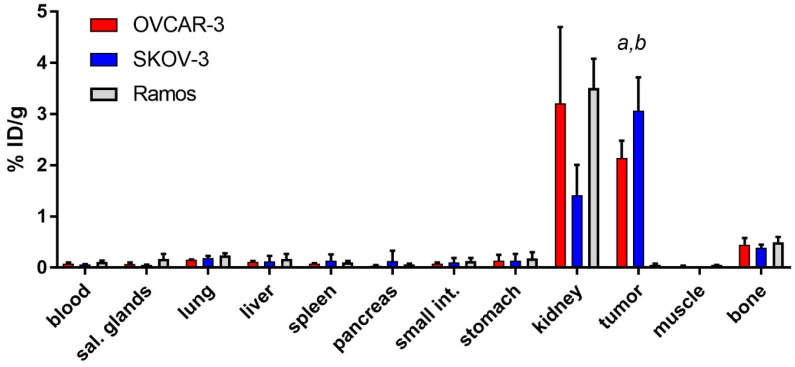
Comparative biodistribution of [^125^I]I-PIB-Ec1 at 6 h pi in Balb/c nu/nu mice bearing EpCAM-expressing OVCAR-3 and SKOV-3 xenografts and EpCAM-negative Ramos xenografts. Data are presented as the mean ± SD from three to six mice. Letters indicate significant differences (*p* < 0.05, one-way ANOVA with Bonferroni’s multiple comparisons test) between: ^a^ tumor uptake in SKOV-3 and Ramos xenografts; ^b^ tumor uptake in OVCAR-3 and Ramos xenografts.

Biodistribution of [^125^I]I-PIB-Ec1 was performed in Balb/c nu/nu mice bearing EpCAM-expressing OVCAR-3 and SKOV-3 xenografts 6 h pi (Figure 5). Biodistribution was characterized by low level of activity retention in majority of normal organs and tissues. The only organ with noticeable activity were kidneys, where the level of activity was comparable to the activity in tumors. No significant (*p* > 0.05, unpaired *t*-test) differences in activity level in normal organs or tissues were observed between OVCAR-3 and SKOV-3 groups, except a small but significant difference in lungs. The uptake of [^125^I]I-PIB-Ec1 in SKOV-3 xenografts was significantly (*p* < 0.05, unpaired *t*-test) higher than in OVCAR-3 xenografts (3.1 ± 0.7 vs. 2.1 ± 0.3 %ID/g).

The activity uptake in EpCAM-negative Ramos (Figure 5) xenografts was significantly (*p* < 0.05, one-way ANOVA with Bonferroni’s multiple comparisons test) lower compared to the uptake in EpCAM-expressing SKOV-3 or OVCAR-3 xenografts.

Low accumulation of activity in normal organs provided high tumor-to-organ ratios in both OVCAR-3 and SKOV-3 xenograft models (Table 2). Due to higher [^125^I]I-PIB-Ec1 uptake in SKOV-3 tumors, significantly (*p* < 0.05, unpaired *t*-test) higher tumor-to-organ ratios were observed in this model. However, ratios of [^125^I]I-PIB-Ec1 uptake in tumor and organs located in peritoneum was high even for mice bearing OVCAR-3 xenografts.

**Figure 6:**
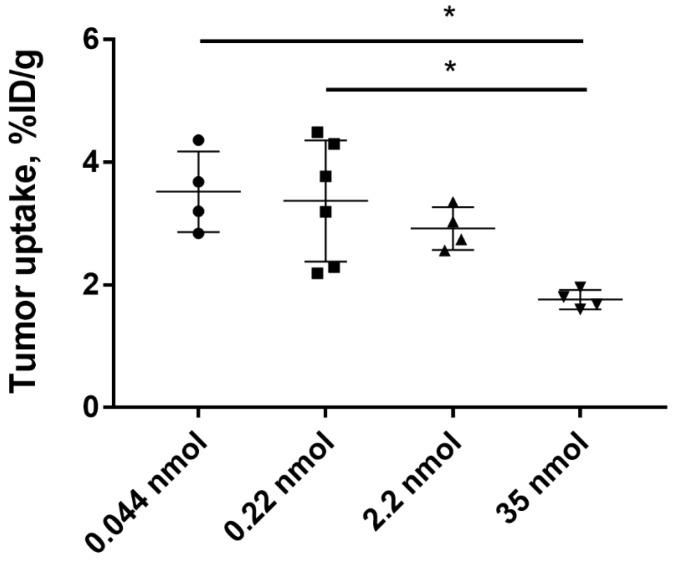
Tumor uptake of [^125^I]I-PIB-Ec1 in Balb/c nu/nu mice bearing SKOV-3 xenografts injected with 0.8, 4, 40, or 640 µg (corresponding to 0.044, 0.22, 22, and 35 nmol) total protein amount 6 h pi. Asterisks show significant differences (*p* < 0.05, one-way ANOVA with Bonferroni’s multiple comparisons test) between the groups.

To investigate the influence of protein dose on tumor uptake of [^125^I]I-PIB-Ec1, Balb/c nu/nu mice bearing SKOV-3 xenografts were injected with 0.8, 4, 40, and 640 µg (corresponding to 0.044, 0.22, 22, and 35 nmol per mouse) of radiolabeled protein and biodistribution was measured 6 h pi. No significant (*p* > 0.05, one-way ANOVA with Bonferroni’s multiple comparisons test) differences in tumor uptake between the groups injected with 0.8, 4, or 40 µg was observed. The tumor uptake of [^125^I]I-PIB-Ec1 in mice injected with 640 µg of Ec1 was significantly (*p* < 0.05, one-way ANOVA with Bonferroni’s multiple comparisons test) lower compared to groups injected with 0.8 or 4 µg (Figure 6).

**Figure 7:**
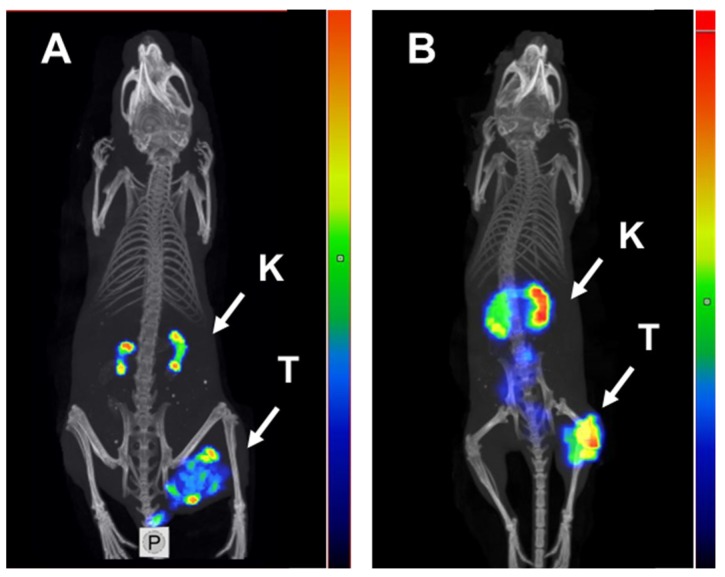
Micro-single photon emission computed tomography (SPECT)/CT imaging of EpCAM expression in Balb/c nu/nu mice bearing (**A**) OVCAR-3 and (**B**) SKOV-3 xenografts using [^125^I]I-PIB-Ec1 6 h pi. K—kidneys, T—tumor.

SPECT/CT imaging using [^125^I]I-PIB-Ec1 in Balb/c nu/nu mice bearing OVCAR-3 and SKOV-3 xenografts at 6 h pi confirmed the results of the biodistribution studies (Figure 7). In both models, low uptake of activity in normal organs, except kidneys, was observed. The level of uptake in kidneys was similar to the uptake in tumors. Radiolabeled [^125^I]I-PIB-Ec1 provided clear visualization of both EpCAM-expressing xenografts.

## 3. Discussion

Our ultimate goal is the development of a therapeutic for targeted systemic treatment of disseminated OC using cytotoxic payload. EpCAM is an attractive target for this purpose due to sufficiently high expression in OC, and DARPins seem to be a promising type of targeting vectors because their small size permits efficient diffusion into tumor extracellular space. However, not all ovarian carcinomas have sufficiently high EpCAM expression [3,4], and there is a risk of overtreatment of patients with tumors expressing low levels of EpCAM. Therefore, we have to co-develop an imaging companion diagnostic for visualization of EpCAM to enable patients’ stratification. Experience with probes based on another type of ESP, affibody molecules, suggests that selection of an optimal labeling strategy is crucial to obtain high imaging contrast and sensitivity [31].

After binding to cell-surface receptors in tumors, radiolabeled proteins are internalized and degraded inside the lysosomal compartment of cells with formation of smaller fragments, radiocatabolites. A similar process takes place after binding to scavenger receptors in excretory organs (kidneys and liver), and, to some extent, after unspecific uptake in other normal tissues. Further fate of activity depends on physicochemical properties of radiocatabolites. Radiocatabolites of residualizing labels (typically charged and/or hydrophilic metal chelates) become trapped inside lysosomes. Radiocatabolites of non-residualizing labels diffuse through the membranes into the extracellular space and the activity is returned back to blood circulation. Direct radioiodination of tyrosine residues in a protein usually results in formation of primary and secondary radiocatabolites that accumulate in organs with expression of Na/I-symporters, such as thyroid, salivary glands, and stomach. On the contrary, radiocatabolites formed after intracellular degradation of indirect labels do not accumulate in these organs and are efficiently removed from the body. Indirect radioiodination of targeting proteins using bifunctional linkers, such as *para*-iodobenzoate (PIB), allows for reduction of activity retention in normal organs and tissues [25,29,32]. If the internalization of a targeting protein by cancer cells is slower than by cells in normal organs and tissues, the use of non-residualizing labels enables high retention of activity in tumors together with its rapid clearance from normal organs and tissues.

We have found that indirect iodination using PIB was the most optimal labeling strategy that enabled high contrast imaging of EpCAM expression in pancreatic cancer in vivo [29]. Based on our previous findings that DARPin Ec1 was slowly internalized by pancreatic BxPC-3 cancer cells and that the use of non-residualizing [^125^I]I-PIB label provided high imaging contrast in pancreatic cancer model, we hypothesized that [^125^I]I-PIB-Ec1 would enable imaging of EpCAM expression in ovarian cancer.

The possibility to use a non-residualizing label depends on internalization rate. Slow internalization of [^125^I]I-PIB-Ec1 by pancreatic cell lines was a precondition for its success as an imaging probe. However, it is not given that the internalization rate would be as slow in the case of ovarian cancer. Activation of genes is dependent on tissue of origin, and an interplay of genes causing internalization of a complex of imaging probes with their molecular target is very difficult to predict a priori. Very different scenarios are possible. Previously, we observed that internalization rate of anti-HER2 affibody molecules was approximately the same for epidermoid and breast cancer cells [33]. However, internalization of radiolabeled HER2-binding ADAPTs was noticeably more rapid by ovarian cancer cells than by breast cancer cells [34]. Internalization rate of anti-HER3 affibody constructs was appreciably higher for pancreatic cancer cells than for prostate cancer cells [35]. Thus, an important result in this study was the demonstration that although internalized fraction of activity was higher for both tested ovarian cancer cell lines (Figure 3), the difference was not dramatic. In vitro binding specificity experiments confirmed that the binding of [^125^I]I-PIB-Ec1 to OVCAR-3 and SKOV-3 ovarian cancer cells was EpCAM-mediated (Figure 2). Affinity measurements using LigandTracer showed high affinity binding in the picomolar range (Figure 3, Table 1), which was in agreement with the previously reported data for [^125^I]I-PIB-Ec1 [27]. 

Slow internalization in vitro is not always translated into slow internalization in vivo [36], because cancer cells in vivo are exposed to a broader spectrum of signaling substances than it is possible to mimic in vitro. However, the results of the biodistribution experiments (Figure 5) showed that uptake of [^125^I]I-PIB-Ec1 in ovarian cancer xenografts (3.1 ± 0.7 and 2.1 ± 0.3 %ID/g at 6 h after injection, for SKOV-3 and OVCAR-3 xenografts, respectively) was in the same range as for pancreatic cancer BxPC-3 xenografts at the same time point (3.2 ± 0.8 %ID/g) [29]. Importantly, in vivo experiments demonstrated specificity of [^125^I]I-PIB-Ec1 uptake in ovarian cancer xenografts in two different ways. Uptake of [^125^I]I-PIB-Ec1 in EpCAM-negative Ramos lymphoma xenografts was much lower (*p* < 0.001) than uptake in EpCAM-positive SKOV-3 and OVCAR-3 xenografts (Figure 5). A significant decrease of uptake of [^125^I]I-PIB-Ec1 in SKOV-3 xenografts after injection of 36 nmol compared to lower doses (Figure 6) demonstrated saturable accumulation, which is a strong indication of its target specificity. The low [^125^I]I-PIB-Ec1 uptake in Ramos lymphoma xenografts shows that EPR effect does not play any role in tumor accumulation. This precludes false-positive findings caused by an unspecific uptake of a tracer in tumors. In both ovarian cancer models, [^125^I]I-PIB-Ec1 demonstrated high tumor-to-organ ratios (Table 2). Particularly important is the high contrast in peritoneal organs, which constitute an anatomical context for ovarian cancer metastases. The high contrast was confirmed by the small animal SPECT/CT imaging (Figure 7). Apparently, iodine-125 has to be substituted by iodine-123 for SPECT or iodine-124 for PET imaging in clinical translation. Another possibility for PET imaging could be to use fluorine-18 labels with non-residualizing properties, e.g., [^18^F]-fluorobenzaldehyde.

Both expression level and accessibility of a molecular target are essential for a successful targeted therapy. It is important that a target saturation effect would not prevent sufficient binding of targeted cytotoxic constructs. Previous studies with anti-EpCAM DARPins fused with truncated Pseudomonas Exotoxin A (ETA’) indicated the injected protein doses necessary for suppression of tumor growth. An injection of 340–500 pmol of Ec4-ETA´´ fusion was sufficient for significant antitumor effect in HT29 colorectal carcinoma and SW2 small cell lung carcinoma xenografts in mice [27]. Significant antitumor effect was observed in HT29 colorectal carcinoma and MDA-MB-468 breast carcinoma xenografts after injection of 350 pmol Ec1-ETA´´ [28]. As no quantitative information about the uptake of DARPin-based agents targeting EpCAM in SKOV-3 tumor model was available, we chose a wide range of concentrations based on our previous experience with other types of scaffold proteins, affibody molecules, and ADAPTs. To observe the maximum level of tumor uptake without saturation, the lowest dose within the handling accuracy was selected. To investigate what dose would cause saturation of tumor uptake and to what extent, doses exceeding the reported therapeutic dose of 500 pmol were chosen. The results of the tumor saturation experiment (Figure 6) demonstrated that such doses of Ec1 can be administered to mice bearing SKOV-3 xenografts without saturating the uptake in tumors. This provides a rationale for dose selection in further studies using therapeutic conjugates of Ec1 with drugs or toxins for targeted therapy. For therapeutic applications, Ec1 could be fused with an albumin-binding domain for extension of half-life in blood and prevention of high renal reabsorption. This approach has previously been applied to develop affibody–drug conjugates and provided extension of survival of mice with HER2-overexpressing tumors without observable toxicities to kidneys or liver [37].

## 4. Materials and Methods

### 4.1. General Materials and Instruments

Iodine [^125^I]NaI was purchased from Perkin Elmer Sverige AB (Sweden). Radiochemical yield and purity were analyzed using instant thin-layer chromatography (iTLC) on iTLC silica gel strips (Varian, Lake Forest, CA, USA). The distribution of activity along strips was measured using a Cyclone storage phosphor system (Packard Instrument Company, Downers Grove, IL, USA) and analyzed by OptiQuant image analysis software (Perkin Elmer, Waltham, MA, USA). Purification of [^125^I]I-PIB-Ec1 was performed by size-exclusion NAP-5 columns (GE Healthcare, UK). Activity in organs and tissues was measured using an automated gamma-spectrometer with a NaI(TI) detector (1480 Wizard, Wallac, Finland). Cell lines used in this study (SKOV-3, OVCAR-3, and Ramos cells) were purchased from the American Type Culture Collection (ATCC) and were cultured under standard conditions in a humidified incubator with 5% CO_2_ at 37 °C, unless stated otherwise. SKOV-3 and Ramos cells were cultured in RPMI medium (Biochrom, Berlin, Germany) containing 10% fetal bovine serum (FBS) (Merck, Darmstadt, Germany), 2 mM L-glutamine, 100 IU/mL penicillin, and 100 µg/mL streptomycin (all from Biochrom, Berlin, Germany). OVCAR-3 cells were cultured in RPMI medium containing 20% FBS, 2 mM L-glutamine, 100 IU/mL penicillin, 100 µg/mL streptomycin, and 0.01 mg/mL bovine insulin (Sigma-Aldrich, St. Louis, MO, USA).

### 4.2. Protein Production and Radiolabeling

The EpCAM-targeting Ec1 DARPin with a H_6_-tag at N-terminus was produced, purified, and characterized as described earlier [27]. Indirect radioiodination of Ec1 using N-succinimidyl-para-(trimethylstannyl)benzoate was performed as described earlier [25,29]. An equal volume of 0.1% acetic acid in water (3.5–35 µL) was added to radioiodine (3.5–35 µL, 9–114 MBq), followed by addition of N-succinimidyl-p-(trimethylstannyl)benzoate (6.5 nmoles, 2.5 µg, 2.5 µL of 1 mg/mL in 5% acetic acid in methanol). The reaction was started by addition of chloramine-T (20 µg, 5 µL, 4 mg/mL in water) and was stopped by addition of sodium metabisulfite (30 µg, 5 µL, 6 mg/mL in water) after 5 min of incubation at room temperature. Then, DARPin Ec1 (3.8 nmoles, 70 µg, 19.5 µL of 3.6 mg/mL in PBS) in 100–120 µL of 0.07 M borate buffer (pH 9.3) was added and incubated at room temperature for 60 min. The radiolabeled conjugate was purified on a NAP-5 column. The labeling yield and purity were determined using radio-iTLC analysis in 4:1 acetone:water system.

### 4.3. Binding Specificity and Cellular Processing Assays

Ovarian cancer cell lines SKOV-3 and OVCAR-3 were used for in vitro studies. The day before the experiment, ca. 10^6^ cells were seeded in 3 cm petri dishes, three dishes per group.

Evaluation of [^125^I]I-PIB-Ec1 binding specificity to EpCAM was performed following a previously described method [29,38]. For saturation of EpCAM receptors, 100-fold excess of non-labeled Ec1 DARPin (200 nM) in cell culture medium was added to one group of cells, equal volume of media only was added to the second group. After 30 min of incubation at room temperature, radiolabeled [^125^I]I-PIB-Ec1 was added to both groups at 2 nM final concentration. After 6 h of incubation at room temperature, the medium was collected, cells were washed, and trypsin was added to detach the cells. The cell suspension was collected and the radioactivity of cells and medium was measured to calculate the percent of cell-bound radioactivity. The data were analyzed using unpaired two-tailed *t*-test.

Cellular retention and processing of [^125^I]I-PIB-Ec1 was studied during continuous incubation using an acid-wash method [38]. Radiolabeled [^125^I]I-PIB-Ec1 (1 nM) was added to cells and incubated for a period of 1, 2, 4, 6, and 24 h. At the designated time point, the media from cells was collected, cells were washed once with serum-free media and were treated with 0.2 M glycine buffer containing 4 M urea (pH 2.0) on ice for 5 min to collect the membrane-bound fraction. Afterwards, the cells were washed with the same buffer and treated with 1 M NaOH for 30 min to collect the fraction containing the internalized compound. The maximum value of cell-associated activity for each cell line was taken as 100% and the other values were normalized to it.

### 4.4. Affinity Measurements Using LigandTracer

The kinetics of [^125^I]I-PIB-Ec1 binding to living SKOV-3 and OVCAR-3 cells was measured using LigandTracer instrument (Ridgeview Instruments, Vänge, Sweden) [39]. After the background measurement, two concentrations of [^125^I]I-PIB-Ec1 (3 and 9 nM) were added to cells to measure the association phase. After that, cell culture media was exchanged to measure the retention in the dissociation phase. Measurements were performed at room temperature. Dissociation constants were calculated using the TraceDrawer Software (Ridgeview Instruments, Vänge, Sweden) based on association and dissociation rates.

### 4.5. Animal Studies

Animal studies were planned and performed following national legislation on protection of laboratory animals and were approved by the local ethics committee for animal research in Uppsala, Sweden (ethical permission C5/16 from 26-02-2016).

For establishment of xenografts, 10^7^ of SKOV-3 and OVCAR-3 cells or 5 × 10^6^ Ramos cells in 100 µL of media were subcutaneously injected in the right hind leg of female Balb/c nu/nu mice. The experiments in mice bearing SKOV-3 and Ramos xenografts were performed 2–3 weeks after implantation. The experiments in mice bearing OVCAR-3 xenografts were performed 7 weeks after implantation. The average mouse weight was 18 ± 2 g. The average tumor weight was 0.2 ± 0.1 g for OVCAR-3 and Ramos, 0.4 ± 0.2 g for SKOV-3. For biodistribution studies all mice were intravenously (i.v.) injected with [^125^I]I-PIB-Ec1 in 100 μL of 1% BSA in PBS per mouse (total protein dose 4 µg, 20 kBq). To investigate the effect of protein dose on tumor uptake, mice bearing SKOV-3 xenografts were injected with [^125^I]I-PIB-Ec1 in 100 μL of 1% BSA in PBS per mouse, total protein doses (0.8, 4, 40, and 640 µg, corresponding to 0.044, 0.22, 22, and 35 nmol per mouse) were adjusted using non-labeled Ec1 DARPin. At 6 h post-injection (pi) of [^125^I]I-PIB-Ec1 mice were received an intraperitoneal injection of ketamine (250 mg/kg) and xylazine (25 mg/kg) and sacrificed by heart puncture. The percentage of injected dose per gram of sample (%ID/g) was calculated.

Whole body SPECT/CT scans of mice bearing SKOV-3 and OVCAR-3 xenografts were performed using nanoScan SPECT/CT (Mediso Medical Imaging Systems, Hungary). Mice were injected with [^125^I]I-PIB-Ec1 (20 μg, 1.2 MBq for SKOV-3, and 6 μg, 2.8 MBq for OVCAR-3). At 6 h pi mice were sacrificed by CO_2_. Images were acquired for 20 min. The parameters of CT scans and SPECT raw data reconstruction were as described in [29]. SPECT raw data were reconstructed using Tera-Tomo™ 3D SPECT reconstruction technology (version 3.00.020.000; Mediso Medical Imaging Systems Ltd.) and CT data were reconstructed using Filter Back Projection in Nucline 2.03 Software (Mediso Medical Imaging Systems Ltd.). Fusion of SPECT and CT files was performed in Nucline 2.03 Software. Images are presented as maximum intensity projections in the RGB color scale.

Statistical analysis. The data from in vitro specificity and cell processing assays are presented as the mean ± standard deviation (SD) (*n* = 3). For statistical analysis GraphPad Prism (version 7.02; GraphPad Software, Inc., La Jolla, CA, USA) was used. Data analysis was performed using an unpaired two-tailed *t*-test with the statistical significance level set at *p* < 0.05. One-way ANOVA with Bonferroni’s multiple comparisons test was applied when more than two data sets were compared.

## 5. Conclusions

A slow internalization of Ec1 DARPin after binding to EpCAM on the surface of ovarian cancer cells determines good cellular retention of activity in the case of non-residualizing labels. Indirectly labeled [^125^I]I-PIB-Ec1 binds to ovarian cell lines with high affinity and specificity. DARPin [^125^I]I-PIB-Ec1 specifically targets ovarian cancer xenografts in vivo. Low retention of [^125^I]I-PIB-Ec1 radiometabolites in normal tissues ensures a high imaging contrast. Results of in vivo saturation experiments suggest that the use of Ec1 DARPin permits delivery of substantial amount of cytotoxic payload to tumors.

## Figures and Tables

**Table 1:** Dissociation equilibrium constants (K*_D_*) for the interaction between [^125^I]I-PIB-Ec1 and OVCAR-3 and SKOV-3 cells.

Cell Line	***K_D_*** **(pM)**
OVCAR-3	35 ± 1
SKOV-3	80 ± 10

**Table 2:** Tumor-to-organ ratios for [^125^I]I-PIB-Ec1 in Balb/c nu/nu mice bearing OVCAR-3 and SKOV-3 xenografts at 6 h pi. Results are presented as average ± SD of four to six mice. ^a^ Significant (*p* < 0.05) difference between OVCAR-3 and SKOV-3 groups (unpaired *t*-test).

Tissue/Organ	OVCAR-3	**SKOV-3**
**Blood**	30 ± 11 ^a^	48 ± 12
**Salivary glands**	36 ± 19 ^a^	59 ± 8
**Lung**	15 ± 4	16 ± 2
**Liver**	20 ± 4 ^a^	45 ± 10
**Spleen**	28 ± 6	40 ± 9
**Pancreas**	57 ± 17 ^a^	102 ± 17
**Small intestine**	30 ± 8	55 ± 16
**Stomach**	20 ± 10 ^a^	42 ± 7
**Kidney**	0.8 ± 0.4 ^a^	2.4 ± 0.8
**Muscle**	71 ± 25	97 ± 28
**Bone**	5 ± 2	8 ± 2

## Abbreviations

ADAPT BSA CT	Albumin-binding domain derived affinity protein Bovine serum albumin Computed tomography
DARPin	Designed ankyrin repeat protein
EpCAM	Epithelial cell adhesion molecule
HER2	Human epidermal growth factor receptor 2
iTLC PBS PET PIB	Instant thin layer chromatography Phosphate buffered saline Positron emission tomography *Para*-iodobenzoate
RGB SPECT	Red, green and blue color scale Single photon emission computed tomography
